# Sustained attention and prediction: distinct brain maturation trajectories during adolescence

**DOI:** 10.3389/fnhum.2015.00519

**Published:** 2015-09-24

**Authors:** Alix Thillay, Sylvie Roux, Valérie Gissot, Isabelle Carteau-Martin, Robert T. Knight, Frédérique Bonnet-Brilhault, Aurélie Bidet-Caulet

**Affiliations:** ^1^UMR Inserm U930, Equipe 1, Centre Universitaire de Pédopsychiatrie, Université François-Rabelais de Tours, CHRU de ToursTours, France; ^2^Inserm CIC 1415, CHRU de ToursTours, France; ^3^Centre Universitaire de Pédopsychiatrie, CHRU de ToursTours, France; ^4^Helen Wills Neuroscience Institute and the Department of Psychology, University of CaliforniaBerkeley, CA, USA; ^5^Inserm, U1028, CNRS UMRS5292, Centre de Recherche en Neurosciences de LyonBron, France

**Keywords:** development, sustained attention, prediction, EEG, ERP

## Abstract

Adolescence is a key period for frontal cortex maturation necessary for the development of cognitive ability. Sustained attention and prediction are cognitive functions critical for optimizing sensory processing, and essential to efficiently adapt behaviors in an ever-changing world. The aim of the current study was to investigate the brain developmental trajectories of attentive and predictive processing through adolescence. We recorded EEG in 36 participants from the age of 12–24 years (three age groups: 12–14, 14–17, 18–24 years) to target development during early and late adolescence, and early adulthood. We chose a visual target detection task which loaded upon sustained attention, and we manipulated target predictability. Continued maturation of sustained attention after age 12 was evidenced by improved performance (hits, false alarms (FAs) and sensitivity) in a detection task, associated with a frontal shift in the scalp topographies of the Contingent Negative Variation (CNV) and P3 responses, with increasing age. No effect of age was observed on predictive processing, with all ages showing similar benefits in reaction time, increases in P3 amplitude (indexing predictive value encoding and memorization), increases in CNV amplitude (corresponding to prediction implementation) and reduction in target-P3 latency (reflecting successful prediction building and use), with increased predictive content. This suggests that adolescents extracted and used predictive information to generate predictions as well as adults. The present results show that predictive and attentive processing follow distinct brain developmental trajectories: prediction abilities seem mature by the age of 12 and sustained attention continues to improve after 12-years of age and is associated with maturational changes in the frontal cortices.

## Introduction

The optimization of sensory processing by the brain, a limited-capacity system, is a key function that alleviates the burden of sensory information to be processed. This optimization is enabled by two complementary processes: (1) attention which prioritizes sensory processing according to task-relevance (Sarter et al., 2001); (2) prediction which facilitates perception based on prior likelihood (Summerfield and Egner, 2009). Among attention mechanisms, sustained attention is defined as the ability to maintain the focus of cognitive activity over time on a given task or stimulation, and is crucial to determine the efficacy of cognitive functions and behaviors (Sarter et al., 2001). Importantly, sustained attention, also referred as tonic alertness (Rueda and Posner, 2013), is thought to underpin other attentional mechanisms such as selective and divided attention as well as general cognitive abilities (Sarter et al., 2001). Predictive processing allows us to employ prior or current information to predict future behavior, and is a key feature of many cognitive functions (Bubic et al., 2010). Predictive processing is far from being a unitary function and comprises several mechanisms such as the generation of prediction based on encoding and memorization of predictive information, and the implementation of prediction via the deployment of preparatory mechanisms resulting in facilitated processing of upcoming events and optimized behaviors. Sustained attention and prediction are thus essential to efficiently adapt behaviors in an ever-changing world and support “proactive” control defined as sustained preparatory mechanisms for upcoming events according to the Dual Mechanisms of Control framework (Braver, 2012).

Interestingly, young children experience difficulties in utilizing predictive information and maintaining that information over a few seconds (Chatham et al., 2009), suggesting a gradual shift, with development, from a reactive posterior stimulus-driven strategy to a proactive anterior top-down strategy in cognitive control (Rubia et al., 2010; Andrews-Hanna et al., 2011; Smith et al., 2011; Munakata et al., 2012; Padilla et al., 2013; Chevalier et al., 2014; Lucenet and Blaye, 2014). Adolescence could be the key period in development for this cognitive shift. Indeed, adolescence is characterized by functional maturation of cognitive processes, in particular of attention and executive functions necessary to generate and use prediction, as observed with fMRI (Rubia et al., 2010) and EEG (Travis, 1998; Segalowitz et al., 2010). These advances in cognitive performance during adolescence are paralleled by neurobiological changes especially within the frontal cortex, which plays a crucial role in sustained attention and prediction processes (Corbetta et al., 2008; Bar, 2009; Summerfield and Egner, 2009). The protracted maturation of the frontal lobes is characterized by gray matter tissue loss and increases in white matter volume (e.g., Yakovlev and Lecours, 1967; Huttenlocher and Dabholkar, 1997; Tau and Peterson, 2009; Giorgio et al., 2010). Therefore, investigating the maturation of prediction and sustained attention capacities during typical development, and especially through adolescence, is crucial to understand the shift in cognitive control strategies and to provide insight in atypical developmental trajectories.

Sustained attention has been widely investigated using neuropsychological tests. However, evidence for the maturational timeline of sustained attention during adolescence is inconsistent, with findings of mature performance by 12-years of age (Lin et al., 1999; Anderson et al., 2001; Kanaka et al., 2008), or of large performance improvement after age 12 (Greenberg and Waldman, 1993; Conners et al., 2003; Li et al., 2009; McAvinue et al., 2012; Tamnes et al., 2012; Boelema et al., 2014). fMRI studies showed a progressive activation increase, through adolescence, in fronto-temporo-parietal regions involved in attention (Rubia et al., 2010; Smith et al., 2011). Brain markers of different mechanisms involved in sustained attention have been identified in studies using electroencephalography (EEG). Sustained attentional preparation can be indexed by the deployment of a centrally distributed event-related potential (ERP), the Contingent Negative Variation (CNV; Segalowitz et al., 1997; Kropp et al., 2001). With growing age, the CNV amplitude over the vertex increases (Timsit-Berthier and Hausmann, 1972; Jonkman, 2006; Hämmerer et al., 2010), and its topographical distribution changes: Tecce (1971) reported an increase in frontal CNV contribution, while Padilla et al. (2013) observed a decrease in parietal CNV contribution beginning at age 12. Moreover, allocation of attentional resources to relevant stimuli can be reflected by the P3 amplitude (Becker and Shapiro, 1980; Johnson, 1986; Peltz et al., 2011). However, there is no consensus on the development of P3 amplitude during adolescence (Sangal et al., 1998; Segalowitz and Davies, 2004; Polich, 2007; Stige et al., 2007).

In adults, we previously defined EEG markers of predictive processing, in a detection task manipulating target predictability (Bidet-Caulet et al., 2012). The P3 amplitude to predictive stimuli was found to index predictive information encoding and maintenance, increase in CNV amplitude to reflect the deployment of anticipatory mechanisms, and the P3 latency to predicted target to serve as a measure of the prediction building and implementation (Bidet-Caulet et al., 2012). The development of the mechanisms supporting prediction during adolescence has been poorly investigated. The encoding of predictive cues has not been examined during adolescence and electrophysiological studies of preparatory mechanisms have found inconsistent findings, with evidence for mature preparation around age 12 (Bender et al., 2005; Jonkman, 2006) or for a still developing preparation during late adolescence (Tecce, 1971; Segalowitz and Davies, 2004; Padilla et al., 2013). Thus, the CNV and P3 appear as good candidates to investigate different mechanisms supporting sustained attention and prediction abilities.

Adolescence is a period associated with protracted maturation of the frontal lobes and could play a key role in the developmental shift from reactive to proactive strategies for cognitive control, and in the maturation of the optimization of sensory processing. However, to our knowledge, no study has attempted to compare the maturation trajectories of sustained attention and prediction through adolescence. We recorded EEG in 36 participants from the age of 12–24 years (three age groups: 12–14, 14–17, 18–24 years) to target development during early and late adolescence, and early adulthood. We chose a visual target detection task (Fogelson et al., 2009b; Bidet-Caulet et al., 2012) which loaded upon sustained attention along a relatively extended period of time, and we manipulated target predictability: subjects either detected random targets or targets preceded by a predictive sequence. ERPs to random standards and targets provided measures of preparatory and resource allocation mechanisms during sustained attention; while during the predictive sequence, encoding/memorization of predictive information and deployment of preparatory mechanisms could be investigated, and the implementation of prediction measured during predictable target processing. Thus, based on this paradigm, the current study investigated the developmental trajectories of behavioral and brain markers of attentive and predictive processing through adolescence. Specifically, we wanted to answer the following questions: (1) when do the mechanisms of preparation and resource allocation supporting attention get mature during adolescence? (2) do the different steps involved in prediction, such as encoding and memorization of predictive information and deployment of preparatory mechanisms, continue to develop through adolescence?

## Materials and Methods

### Subjects

Thirty six healthy volunteers (29 men) aged from 12 to 24 years were recruited. All the subjects had normal or corrected-to-normal vision, no history of psychiatric or neurological problems and they were not taking any drug. The Ethics Committee of the University Hospital of Tours approved the protocol. Written informed consent was obtained from the parents and adults and assent from the adolescents.

Subjects were assigned to three subsamples: 12–14 (*N* = 11, 10 men, 12 years, 2 months to 13 years, 11 months, mean ± Standard Error of the Mean = 13 years, 2 months ± 2 months), 14–17 (*N* = 13, 9 men, 14 years, 6 months to 16 years, 10 months, mean ± SEM = 15 years, 8 months ± 2 months), and 18–24 (*N* = 12, 10 men, 18 years to 23 years, 10 months, mean ± SEM = 20 years, 7 months ± 7 months).

### Stimuli and Tasks

**Figure 1:**
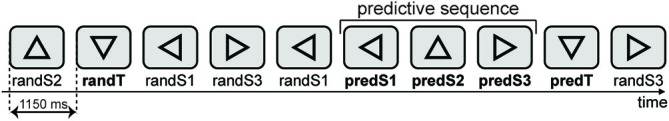
**Stimuli were presented centrally on a computer.** Stimuli consisted of 15% of targets (downward-facing triangle) and 85% of equal amounts of three types of standards: triangles facing left, upward, or right. A target could be a random target (randT) preceded by a non-informative context (random sequence of stimuli) or a predicted target (predT) preceded by an informative context, i.e., a three-stimulus predictive sequence (leftward-, upward- and rightward-facing triangles). Triangles of the predictive sequence are labeled as predS1, predS2 and predS3 stimuli, whereas the corresponding triangles outside the predictive sequence are labeled as randS1, randS2 and randS3, for leftward-, upward- and rightward-facing triangles, respectively.

Subjects sat in a chair in a sound-attenuated room, 94 cm in front of a 19-inch PC screen. The experimenters and computers delivering the visual stimuli and recording the EEG were located in a separate room. We used a paradigm designed to investigate predictive context processing adopted from Fogelson et al. (2009b). Visual stimuli were presented centrally on a computer screen and subtended 3° of visual angle (Figure 1). Each stimulus was presented for 150 ms followed by a 1000 ms blank-screen interstimulus interval.

Stimuli consisted of 15% of targets (downward-facing triangle) and 85% of equal amounts of three types of standards: triangles facing left, upward, or right. A target could be a random target (randT) preceded by a non-informative context (random sequence of stimuli) or a predicted target (predT) preceded by an informative context, i.e., a three-stimulus predictive sequence (leftward-, upward- and rightward-facing triangles). Triangles of the predictive sequence are labeled as predS1, predS2 and predS3 stimuli, whereas the corresponding triangles outside the predictive sequence are labeled as randS1, randS2 and randS3, for leftward-, upward- and rightward-facing triangles, respectively.

Participants were instructed to press a button with the dominant-hand index finger in response to target stimuli (downward-facing triangles) and to look for the predictive sequence. Subjects first performed a brief training session to ensure they were able to detect the target accurately. Subjects were then introduced to the predictive sequence before the recordings began and were aware that it would be 100% predictive of a target, but that targets would also appear randomly throughout the block.

In each block (approximately 2.3 min long), a total of 127 stimuli (11 randTs, 28 randS1, 28 randS2, 28 randS3, 8 predTs, 8 predS1, 8 predS2, and 8 predS3) were presented each for 150 ms with an interstimulus interval of 1 s. 33 subjects performed 15 blocks, leading to a total of 165 randTs, 420 randS1, 420 randS2, 420 randS3, 120 predTs, 120 predS1, 120 predS2, and 120 predS3, for each participant. The duration of the entire test session including practice was approximately of 45 min. Three subjects (2 subjects in the 12–14 year-old group and 1 subject in the 14–17 year-old group) did not wish to continue the task beyond 10 blocks due to fatigue, leading to a total of 110 randTs, 280 randS1, 280 randS2, 280 randS3, 80 predTs, 80 predS1, 80 predS2, and 80 predS3, for each participant. The stimulus presentation and response recordings were controlled using Presentation software (Neurobehavioral Systems, Albany, CA, USA).

### Electroencephalography Recording

EEG was recorded from 64 electrodes using ActiveTwo system (Biosemi, The Netherlands). Vertical eye movements were monitored using electrodes placed above and below the left eye. The signal was recorded with a sampling frequency of 512 Hz and filtered at 0–104 Hz. Data were re-referenced offline to the average potential of the two earlobe electrodes.

### Electroencephalography Data Analysis

EEG analyses were performed on standard and target visual stimuli embedded or not embedded in the predictive sequence. We excluded from further analysis: trials corresponding to standards after a target, standards before or after a button press, a randS2 standard preceded by a randS1 standard but not followed by a randS3 standard (as it is a potential predS2 standard), missed targets, and targets preceded by less than three standards. Eye-movement artifacts were detected using independent component analysis (ICA) and were selectively removed via the inverse ICA transformation. Only 1 or 2 independent components were removed in each subject to clean the data. In six subjects, flat or excessively noisy signals at one or two electrodes were replaced by their values interpolated from the remaining electrodes using spherical spline interpolation (Perrin et al., 1989). Trials contaminated with excessive muscular activity in the (−700; 700 ms) time-window relative to stimulus onset were also excluded.

As the number of trials for stimuli embedded in the predictive sequence was lower than for the other stimuli, we equalized the number of trials within each pair of to-be-compared stimuli by random selection, for each participant. On average across participants, we obtained mean ± Standard Error of the Mean (SEM) 75 ± 2, 92 ± 3, 91 ± 3, and 80 ± 3 clean trials for randS1/predS1, randS2/predS2, randS3/predS3 and randT/predT pairs, respectively, for each participant.

### Event-Related Potential Analysis

We averaged single trials, locked to stimulus onset, separately for each of the eight stimulus categories (randS1, randS3, randS2, randT, predS1, predS2, predS3, predT). The resulting ERPs were digitally band-pass filtered between 0.5 and 30 Hz. For post-stimulus analysis, ERPs were corrected with a −100 to 0 ms baseline before stimulus onset. For pre-stimulus analysis, ERPs were not baseline corrected.

### Statistical Analysis

Analyses of variance (ANOVAs) were used with age group (12–14, 15–17 and 18–24 years) as the between-subject factor to assess the effect of age on different processes. *F* values, probability levels, effect sizes (partial eta squared η_p_ and statistical power (at *α* = 0.05) were provided. When necessary, ANOVAs results were corrected with the Greenhouse-Geisser procedure (epsilon and corrected *p* are reported). Significant main effects obtained with ANOVAs were further examined using *post hoc* randomization tests (Edgington, 1995) for inter-groups comparisons. Randomization consisted of: (1) the random constitution of the two samples to compare; (2) the sum of squared sums of values in the two obtained samples; and (3) the computation of the difference between these two statistic values. We performed 10,000 such randomizations to obtain an estimate of the distribution of this difference under the null hypothesis. This distribution was then compared to the actual difference between the values in the two conditions. Relations between behavioural, neurophysiological measures, and age were assessed with Pearson correlation analyses.

#### Statistical Analysis of Behavioral Data

A button press within the interval of 100–1100 ms after a target onset was considered as a correct response, and a press after a standard (randS1, randS2 or randS3) was counted as a false alarm (FA). Reaction times (RT) were computed for correct trials, only.

Behavioral measures of sustained attention to stimuli outside the predictive sequences were considered. The percentage of hits was computed as the percentage of correct responses to randTs, the percentage of FAs as the percentage of FAs outside the predictive sequence, and the sensitivity as *d*′ = *z*-score (% of correct responses to randTs) − *z*-score (FAs outside the predictive sequence), which takes into account the FA rate to correct for response bias. The effect of age on these measures was assessed using ANOVAs with age group (12–14, 15–17 and 18–24 years) as the between-subject factor.

The effect of predictability on behavioral measures was investigated for the % of hits and RT to randTs and predTs using repeated-measure analyses of variance (rmANOVAs) with age group (12–14, 15–17 and 18–24 years) as the between-subject factor and with predictability (predT vs. randT) as the within-subject factor.

#### Statistical Analysis of Event-Related Potentials

To examine sustained attention as a function of age groups, we analyzed the CNV and P3 responses to all random standard stimuli (averaged ERPs of randS1, randS2 and randS3; standards), and to the random targets (randTs), separately. CNV amplitudes were computed as the mean voltage in the −150–0 ms time-window on electrodes Fz, Cz and Pz separately. P3 amplitudes were computed as the mean voltage in the 350–600 ms time-window on electrodes Fz, Cz and Pz separately. These measures were submitted to rmANOVAs with age group as the between-subject factor and with electrode as the within-subject factor to investigate amplitude effects according to age groups. To explore difference in topographies according to age, the CNV and P3 measures were first normalized to avoid any bias from amplitude effect using a division of the value at each electrode by the norm of the vector in electrode space for each subject (McCarthy and Wood, 1985), and then submitted to rmANOVAs with age group as the between-subject factor and with electrode as the within-subject factor. Significant age group by electrode interaction effects were further explored by computing *post hoc* rmANOVAs for each electrode separately.

Predictive processing according to age was examined by comparing ERPs to the same physical stimuli embedded (predictive stimuli) or not embedded (non-predictive) in the predictive sequence, i.e., we compared predS2 with randS2, predS3 with randS3, and predT with randT. No difference was predicted and none was observed between predS1 and randS1 as participants did not know at that time if the stimulus was part of the predictive sequence or not. CNV amplitudes were computed as the mean voltage in the −150–0 ms time-window on Cz, and were submitted to rmANOVAs with age group as the between-subject factor and with predictability as the within-subject factor. The amplitudes of the P3 to standard stimuli were computed as the mean voltage in the 350–600 ms time-window on Cpz. In response to targets, the latency and the amplitude of the P3 maximum peak in the 250–750 ms time-window on Cpz were extracted. These P3 measures were submitted to rmANOVAs with age group as the between-subject factor and with predictability as the within-subject factor. The selection of electrodes and time-windows of interest was based on results in previous EEG studies (Fogelson et al., 2009b, 2010; Bidet-Caulet et al., 2012).

#### Statistical Analysis of the Relations Between Behavioral Data and Event-Related Potentials

Percentage of hits to randTs, % of FAs, and *d*′ were considered as behavioural measures of sustained attention, and the difference in RTs between randTs and predTs was computed as a behavioural measure of prediction abilities. Relations between these behavioural measures and ERP values were assessed with Pearson correlation analyses.

The ELAN software package was used for visualization and analysis of EEG and ERP (Aguera et al., 2011). STATISTICA v10 (StatSoft, Inc) software was used for ANOVAs and correlation analyses. Randomisation tests were performed using custom MATLAB R2010b (MathWorks, Inc) programs.

## Results

### Sustained Attention Processes

#### Behavioral Results

**Figure 2:**
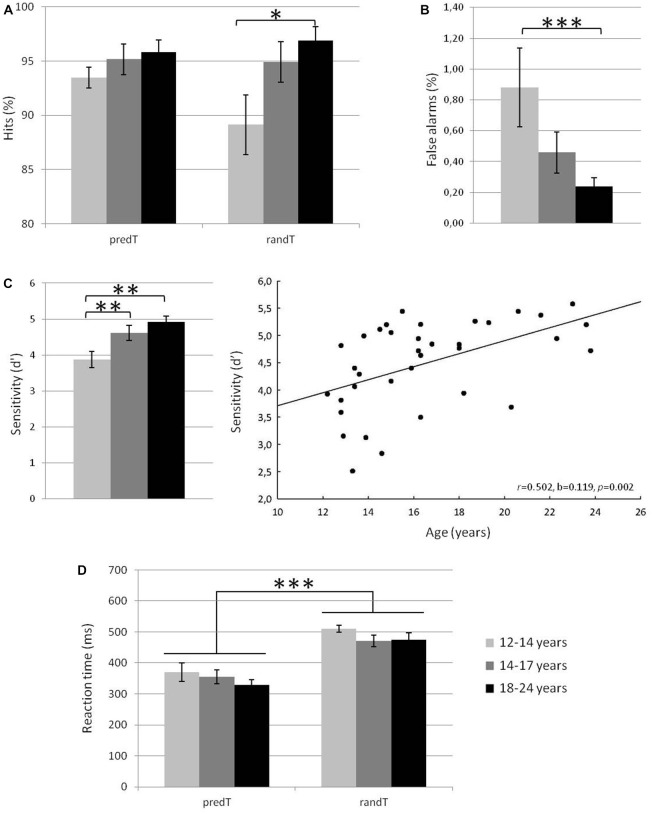
**Mean % of hits for predicted and random targets in the three age groups (A), % of false alarms (FAs) in the three age groups (B), sensitivity *d*′ in the three age groups and plotted against age in years (C) and reaction times (RTs) in ms for predicted and random targets in the three age groups (D).** Error bars: standard errors of mean. Significant differences are indicated by asterisks: *p ≤ 0.05, **p ≤ 0.01, ***p ≤ 0.001.

Behavioral results are displayed in Figure 2. A main effect of age group was significant for % of hits to randTs, % of FAs, and *d*′ (*F*_(2,33)_ = 3.84, *p* = 0.032, η_p_ = 0.189, power = 0.656, *F*_(2,33)_ = 3.85, *p* = 0.031,η_p_ = 0.189, power = 0.657, or* F*_(2,33)_ = 6.66, *p* = 0.004, η_p_ = 0.288, power = 0.887; respectively). *Post hoc* analyses revealed that 12–14 year-olds detected less randTs and made more FAs than 18–24 year-olds (*p* = 0.018 or *p* = 0.001, respectively), and presented a smaller *d*′ compared to 14–17 year-olds and 18–24 year-olds (*p* = 0.011 and *p* = 0.003, respectively). All the other effects were not significant (*p* > 0.081). The % of hits to the randTs and d’ were positively correlated with age (*r* = 0.393, *b* = 0.869, *p* = 0.018, or *r* = 0.502, *b* = 0.119, *p* = 0.002; respectively). The % of FAs was negatively correlated with age (*r* = −0.401, *b* = −0.072, *p* = 0.015). The effect of age group on RTs to randTs was not significant (*F*_(2,33)_ = 1.31, *p* = 0.284, η_p_ = 0.07). In summary, increased age was associated with increased % of hits, reduced % of FAs and increased *d*′, with a more dramatic increase of the *d*′ from early to late adolescence.

#### Event-Related Potential Results

To explore sustained attention according to age, we analyzed the CNV and P3 responses to all random standard stimuli, and to random targets (randTs), separately, using rmANOVAs with electrode and age group as factors.

**Figure 3:**
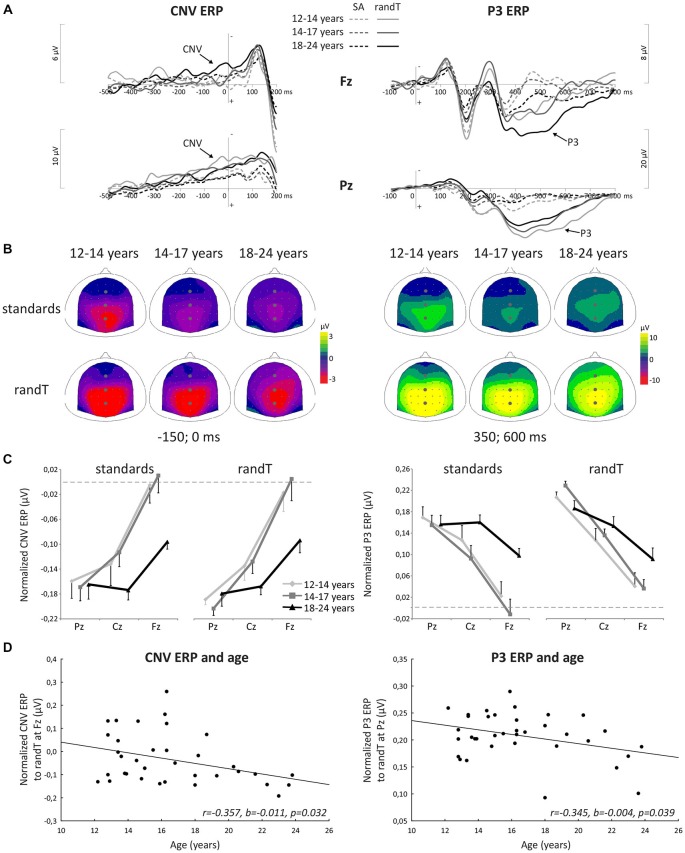
**Attentional processes. (A)** Grand-average ERP waveforms band-pass filtered between 0.5 and 30 Hz, at the Fz and Pz electrodes. **(B)** Scalp topographies (top views) of the mean ERP (CNV and P3) values in the −150–0 ms and 350–600 time-windows. The gray dots indicate the position of Fz, Cz and Pz electrodes. **(C)** Normalized CNV and P3 amplitudes (microvolts) at Fz, Cz and Pz electrodes. Data for standards (left) and randTs (right) are shown for the three age groups. Error bars: standard errors of mean. **(D)** Normalized CNV amplitude (microvolts) preceding randTs at Fz plotted against age in years. Normalized P3 amplitude (microvolts) to randTs at Pz plotted against age in years. The solid lines represent the linear regressions.

CNV amplitude preceding both standards and randTs displayed a significant effect of electrode (*p* < 0.001) and a significant electrode × age group interaction (*p* < 0.044; Table 1). To further understand these differences in topographies, normalized CNV amplitudes were compared. Normalized CNV amplitudes preceding standards or randTs displayed a significant effect of electrode (*p* < 0.001) and a significant electrode × age group interaction (*p* < 0.046; Table 1). This electrode × age group interaction was explained by an effect of age group at Fz, only, significant for standards (*F*_(2,33)_ = 5.86, *p* = 0.007, η_p_ = 0.262, power = 0.842), and nearly reaching significance for randTs (*F*_(2,33)_ = 3.15, *p* = 0.056, η_p_ = 0.160, power = 0.565). *Post hoc* analyses revealed that CNV normalized amplitude at Fz preceding standards and randTs were larger in 18–24 year-olds compared to 14–17 year-olds (*p* = 0.002, or *p* = 0.023; respectively) and compared to 12–14 year-olds (*p* = 0.003, or *p* = 0.047; respectively). No significant differences were found between 12–14 year-olds and 14–17 year-olds on the CNV normalized amplitude at Fz preceding standards and randTs (*p* > 0.644). Moreover, significant negative correlations were found between age and the CNV normalized amplitude at Fz preceding standards and randTs (*r* = −0.357, *b* = −0.010, *p* = 0.032, or *r* = −0.357, *b* = −0.011, *p* = 0.032, respectively; Figure 3).

**Table 1:** **Effect of electrode and age group on the ERP amplitudes**.

	Age group			Electrode					Electrode × age group				
	*F*	*p*	η_p_	*F*	ε	*p*	η_p_	Power	*F*	ε	*p*	η_p_	Power
CNV ERP to standards	0.54	0.588	0.032	32.67	0.586	<0.001	0.497	1.000	3.21	0.586	0.044	0.163	0.801
CNV ERP to randTs	0.17	0.845	0.074	45.28	0.589	<0.001	0.578	1.000	3.95	0.589	0.022	0.193	0.884
Normalized CNV ERP to standards	2.12	0.136	0.114	56.17	0.653	<0.001	0.630	1.000	3.28	0.653	0.035	0.166	0.811
Normalized CNV ERP to randTs	1.70	0.198	0.093	57.91	0.609	<0.001	0.637	1.000	3.12	0.609	0.046	0.159	0.788

P3 ERP to standards	1.32	0.280	0.074	26.79	0.585	<0.001	0.448	0.999	4.37	0.585	0.015	0.209	0.916
P3 ERP to randTs	0.18	0.834	0.076	89.85	0.765	<0.001	0.731	1.000	4.54	0.765	0.006	0.216	0.927
Normalized P3 ERP to standards	2.98	0.065	0.153	49.22	0.626	<0.001	0.599	1.000	3.45	0.626	0.032	0.173	0.832
Normalized P3 ERP to randTs	0.66	0.525	0.038	73.96	0.782	<0.001	0.691	1.000	2.89	0.782	0.042	0.149	0.752

Results of the rmANOVAs on ERP mean amplitudes with electrode as within-subject factor and age group as between-subject factor. Statistical values (F, ε, p, ${\text{η}}_{p}^{2}$ and power) of electrode and age group main effects, and of electrode by age group interaction effect. Significant effects are highlighted in gray.

In summary, older age was associated with a larger (more negative) frontal contribution to the CNV preceding standards and randTs, with a more dramatic increase from late adolescence to early adulthood.

P3 amplitude in response to standards or randTs displayed a significant effect of electrode (*p* < 0.001) and a significant electrode × age group interaction (*p* < 0.015; Table 1). Normalized P3 amplitudes were compared to further understand these differences in topographies. Normalized P3 amplitude in response to standards or randTs displayed a significant effect of electrode (*p* < 0.001) and a significant electrode × age group interaction (*p* < 0.042; Table 1). This electrode × age group interaction was explained by a significant effect of age group to standards at Fz only (*F*_(2,33)_ = 5.59, *p* = 0.008, η_p_ = 0.253, power = 0.823), and by a significant effect of age group to randTs at Pz only (*F*_(2,33)_ = 3.81, *p* = 0.032, η_p_ = 0.187, power = 0.652). *Post hoc* analyses revealed that P3 normalized amplitude at Fz to standards were larger in 18–24 year-olds compared to 14–17 year-olds (*p* = 0.015) and compared to 12–14 year-olds (*p* = 0.016). No difference was found between 12–14 year-olds and 14–17 year-olds on the P3 normalized amplitude at Fz to standards (*p* = 0.400). P3 normalized amplitude at Pz to randTs were smaller in 18–24 year-olds compared to 14–17 year-olds (*p* = 0.016). No differences were found between 12–14 year-olds and 14–17 year-olds, and between 12–14 year-olds and 18–24 year-olds on the P3 normalized amplitude at Pz to randTs (*p* > 0.103). Moreover, age was found to significantly correlate with the P3 normalized amplitude at Pz to randTs (*r* = −0.345, *b* = −0.004, *p* = 0.039; Figure 3). The correlation between age and the P3 normalized amplitude at Fz to standards nearly reached significance (*r* = 0.302, *b* = 0.008, *p* = 0.073).

In summary, older age was associated with a larger frontal contribution to the P3 response to standards, with a more dramatic increase from late adolescence to early adulthood.

#### Correlations with Behavioral Measures of Sustained Attention

**Figure 4:**
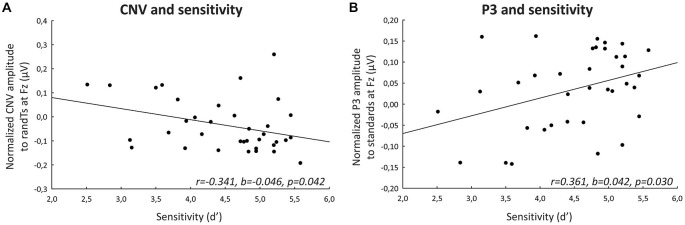
**Correlations with behavioral measures of sustained attention. (A)** Normalized CNV ERP amplitudes (microvolts) preceding randTs at Fz plotted against sensitivity *d*′. **(B)** Normalized P3 ERP amplitudes (microvolts) preceding standards at Fz plotted against sensitivity *d*′. The solid lines represent the linear regressions.

Larger (more negative) CNV normalized ERP amplitudes to randTs (but not to standards, *p* = 0.242) at Fz correlated with greater *d*′ (*p* = 0.042; Figure 4). Larger P3 normalized ERP amplitudes to standards at Fz also correlated with greater *d*′ (*p* = 0.029; Figure 4). The amplitude of P3 normalized ERPs to randTs at Pz was not correlated with *d*′ (*p* = 0.421; Table 2). In summary, increases in CNV and P3 frontal normalized ERP amplitudes were correlated with greater sensitivity.

**Table 2:** **Correlations with behavior**.

		*d′*		
		*r*	*b*	*p*
Normalized CNV ERP to standards	Fz	−0.200	−0.024	0.242
Normalized CNV ERP to randTs	Fz	−0.341	−0.046	0.042
Normalized P3 ERP to standards	Fz	0.361	0.042	0.030
Normalized P3 ERP to randTs	Pz	−0.138	−0.007	0.421

Statistical values (r, b and p) of Pearson correlations between sensitivity (d’) and ERP mean amplitudes. Significant effects are highlighted in gray.

### Predictive Processing

#### Behavioral Results

Behavioral results are displayed in Figure 2. No effect of predictability (*F*_(1,33)_ = 1.43, *p* = 0.240, η_p_ = 0.041) was found on the % of hits to predTs and randTs. The effect of age group (*F*_(2,33)_ = 3.27, *p* = 0.051, η_p_ = 0.165), and the predictability × age interaction (*F*_(2,33)_ = 2.56, *p* = 0.092, η_p_ = 0.134) were close to significance. These effects were driven by a main effect of age group that was significant for the % of hits to randTs (see previous section) but not for the % of hits to predTs (*F*_(2,33)_ = 0.97, *p* = 0.391, η_p_ = 0.055).

The RT to predTs were significantly shorter than those to randTs (*F*_(1,33)_ = 91.23, *p* < 0.001, η_p_ = 0.734, power = 1.000). No effect of age group (*F*_(2,33)_ = 1.24, *p* = 0.302, η_p_ = 0.070), nor predictability × age interaction (*F*_(2,33)_ = 0.44, *p* = 0.645, η_p_ = 0.026) was found significant.

#### Event-Related Potential Results

To investigate predictive processing according to age, we compared the CNV and P3 ERPs to the same physical stimuli embedded or not embedded in the predictive sequence, i.e., we compared predS2 with randS2, predS3 with randS3, and predTs with randTs using rmANOVAs with predictability and age group as factors.

**Figure 5:**
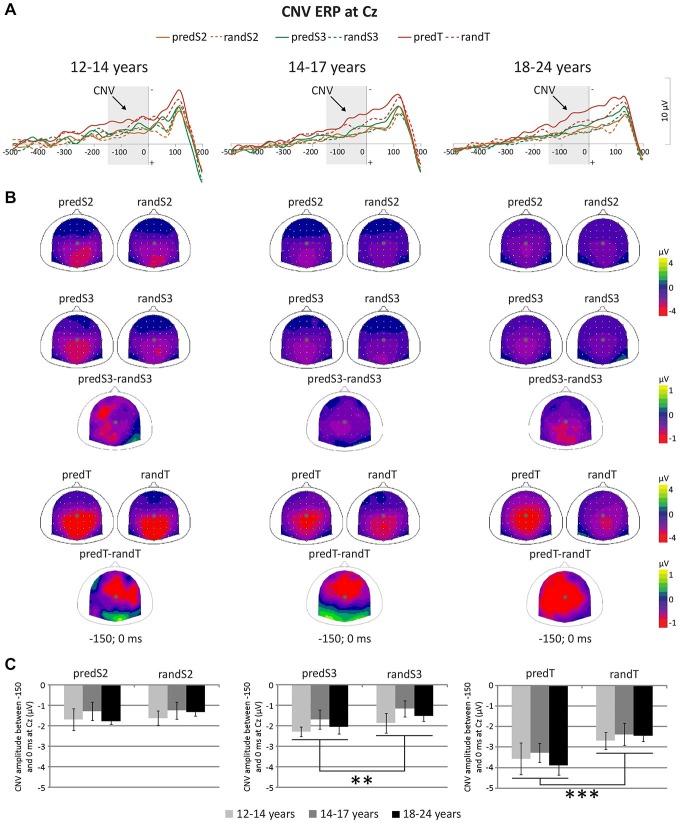
**Predictive processing: CNV ERP. (A)** Grand-average non-baseline-corrected ERP waveforms band-pass filtered between 0.5 and 30 Hz, at the Cz electrode. **(B)** Scalp topographies (top views) of the mean CNV ERP for each pair of predictive stimulus and its non-predictive analog, and for the difference between predS3 and randS3 and between predTs and randTs in the −150–0 ms time-window. The gray dots indicate the position of Cz. **(C)** CNV amplitude between −150 and 0 ms in μV at Cz for predS2 and randS2, for predS3 and randS3, and for predicted and random targets. Error bars: standard errors of mean. Significant differences are indicated by asterisks: **p ≤ 0.01, ***p ≤ 0.001.

A significant effect of predictability was found on CNV amplitude preceding targets (*F*_(1,33)_ = 23.57, *p* < 0.001, η_p_ = 0.417, power = 0.997), and preceding S3 (*F*_(1,33)_ = 9.24, *p* = 0.005, η_p_ = 0.219, power = 0.839), but not preceding S2 (*F*_(1,33)_ = 1.56, *p* = 0.220, η_p_ = 0.045; Figure 5). All the other effects were not significant. These significant effects corresponded to an enhancement of the CNV amplitude before the S3 standard stimuli and targets with predictability increase.

**Figure 6:**
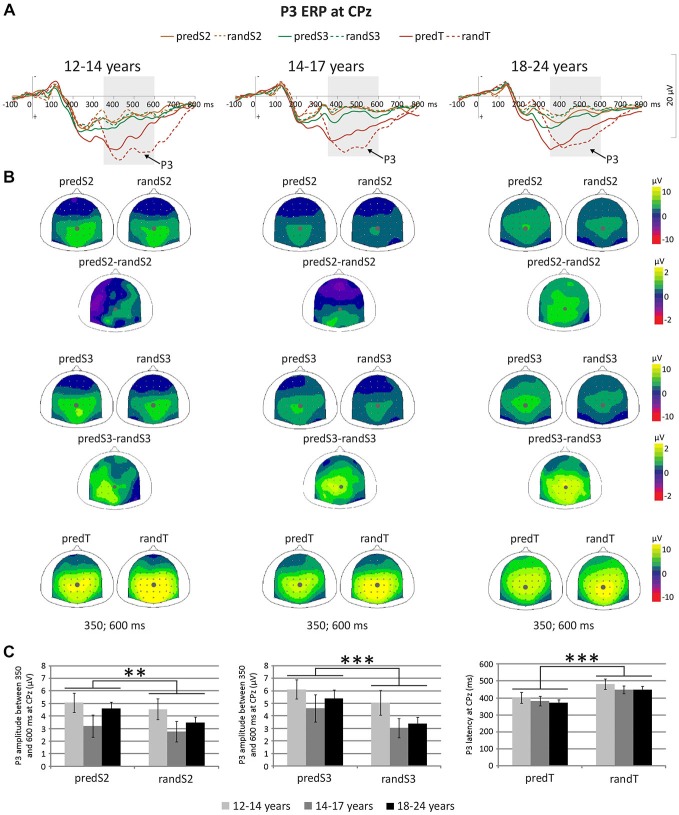
**Predictive processing: P3 ERP. (A)** Grand-average ERP waveforms band-pass filtered between 0.5 and 30 Hz, at the CPz electrode. **(B)** Scalp topographies (top views) of the mean P3 ERP for each pair of predictive stimulus and its non-predictive analog, and for the difference between predS2 and randS2 and between predS3 and randS3 in the 350–600 ms time-window. The gray dots indicate the position of the Cpz electrode. **(C)** P3 amplitude between 350 and 600 ms in μV at CPz for predS2 and randS2, and for predS3 and randS3. Maximum P3 latency in ms at CPz for predicted and random targets. Error bars: standard errors of mean. Significant differences are indicated by asterisks: **p ≤ 0.01, ***p ≤ 0.001.

P3 amplitude to S2 and S3 displayed a significant effect of predictability (*F_(_*_1, 33)_ = 11.87, *p* = 0.002,η_p_ = 0.265, power = 0.917; or *F*_(1, 33)_ = 22.56, *p* < 0.001, η_p_ = 0.406, power = 0.996, respectively), but no effect of age group (*F*_(2,33)_ = 1.63, *p* = 0.212, η_p_ = 0.090; or *F*_(2, 33)_ = 1.29, *p* = 0.288, η_p_ = 0.073, respectively), nor significant predictability × age group interaction (*F*_(2, 33)_ = 1.16, *p* = 0.325, η_p_ = 0.066; or *F*_(2, 33)_ = 0.64, *p* = 0.536, η_p_ = 0.037, respectively; Figure 6). These effects corresponded to an enhancement of the P3 amplitude to standard stimuli with predictive value for both S2 and S3 standard stimuli.

There was no effect of age group on the maximum P3 amplitude at CPz to targets (*F*_(2,33)_ = 1.28, *p* = 0.292, η_p_ = 0.072), nor effect of predictability (*F*_(1,33)_ = 1.83, *p* = 0.185, η_p_ = 0.052), nor predictability × age group interaction (*F*_(1,33)_ = 0.44, *p* = 0.650, η_p_ = 0.026). The P3 ERP to predTs was found earlier in latency than to randTs at CPz (*F*_(1,33)_ = 19.27, *p* < 0.001, η_p_ = 0.369, power = 0.989). No effect of age group (*F*_(2,33)_ = 0.62, *p* = 0.542, η_p_ = 0.036), nor predictability × age group interaction (*F*_(2,33)_ = 0.06, *p* = 0.946, η_p_ = 0.003) was found significant on the P3 latency. Irrespective of age, P3 latency was shortened to predTs.

#### Correlations with a Behavioral Measure of Prediction (Figure 7)

**Figure 7:**
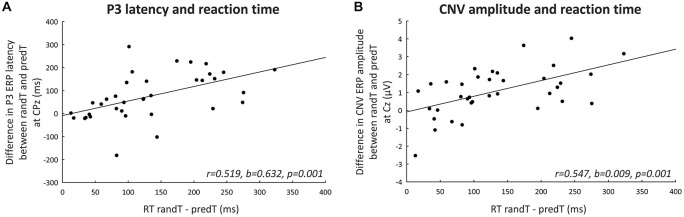
**Correlations with a behavioral measure of prediction. (A)** Difference in maximum P3 ERP latency (ms) between randTs and predTs at CPz plotted against the difference in reaction time between randTs and predTs (ms). **(B)** Difference in CNV ERP amplitude (microvolts) between randTs and predTs at Cz plotted against the difference in reaction time between randTs and predTs (ms). The solid lines represent the linear regressions.

Predictability effect on the amplitude of the CNV preceding targets (difference in CNV amplitude between randTs and predTs) at Cz was correlated with the predictability benefit in reaction time (difference in reaction time between randTs and predTs; *r* = 0.547, *b* = 0.009, *p* = 0.001). Amplitude enhancement benefitted RT.

Predictability effect on the latency of the P3 to targets (difference in P3 latency between randTs and predTs) at CPz was found correlated with the predictability benefit in reaction time (difference in reaction time between randTs and predTs; *r* = 0.519, *b* = 0.632, *p* = 0.001). The larger the latency reduction, the larger the benefit in reaction time.

## Discussion

We provide novel evidence supporting distinct brain maturation trajectories of sustained attention and prediction during adolescence. Continued maturation of sustained attention after age 12 was evidenced by improved performance in a detection task and a frontal shift in P3 and CNV topographies with increasing age. In contrast, predictive processing appeared to be mature at age 12, with all ages showing similar benefits in reaction time, increases in P3 amplitude (indexing predictive value encoding and memorization), increases in CNV amplitude (corresponding to the deployment of preparatory mechanisms) and reduction in target-P3 latency (reflecting efficient prediction building and implementation).

### Maturation of Sustained Attention

We observed improved performance from age 12, with more accurate target detection, better sensitivity and fewer FAs. This pattern of performance decrements in 12–14 year olds, in particular the larger number of omissions, argues for inattention (lapses of sustained attention) rather than for impulsivity.

Previous results on the late maturation of behavioral measures of sustained attention are inconsistent. Using different target detection tasks, reduction in the number of omission and commission (FAs) errors, reduced RT, or improved perceptual sensitivity to targets were found from age 12 to 16 (Greenberg and Waldman, 1993; Conners et al., 2003; Li et al., 2009; McAvinue et al., 2012; Tamnes et al., 2012; Boelema et al., 2014); whereas stable performances were observed in the same age range by others (Lin et al., 1999; Anderson et al., 2001). Though changes in the behavioral measures of sustained attention are more drastic before age 12 (Greenberg and Waldman, 1993; Lin et al., 1999; Luna et al., 2004; Li et al., 2009); the present findings, in agreement with previous studies (Greenberg and Waldman, 1993; Conners et al., 2003; Li et al., 2009; McAvinue et al., 2012; Tamnes et al., 2012; Boelema et al., 2014), suggest that sustained attention is still immature at age 12.

The present electrophysiological results, showing immature CNV and P3 topographies in early and late adolescents, are consistent with a later maturation of sustained attention. More precisely, our results suggest that attentional preparation indexed by the CNV (Tecce, 1971; Segalowitz and Davies, 2004; Padilla et al., 2013) and allocation of attentional resources reflected by the P3 (Segalowitz and Davies, 2004; Stige et al., 2007) are both immature and involved in the continued development of sustained attention after age 12. ERP results show an increase of the frontal contribution to CNV topography with older age, consistent with Tecce (1971), and an increase of the frontal and a decrease of the parietal contributions to P3 with older age, in agreement with previous studies (Courchesne et al., 1978; Mueller et al., 2008). This frontal shift through adolescence is consistent with the proposal of a move in strategy from a posterior stimulus-driven orienting network to engagement of an anterior top-down attention network (Rubia et al., 2010; Smith et al., 2011; Padilla et al., 2013) and a shift from a reactive to a proactive cognitive control (Andrews-Hanna et al., 2011; Munakata et al., 2012; Chevalier et al., 2014; Lucenet and Blaye, 2014). Importantly, sensitivity to targets increases with frontal ERP contribution, suggesting a link between frontal maturation and sustained attention capacities. Several studies provided compelling evidence for a crucial role of the frontal cortex in attention control (e.g., Knight et al., 1995; Sarter et al., 2001; Corbetta and Shulman, 2002). Immature activation of frontal areas mediating attentional processes through adolescence suggests that improvements in these skills could correspond to anatomical maturation of frontal areas. In fact, concurrent with cognitive development are important brain maturational changes that continue into early adulthood, including myelination, synaptic pruning and dopaminergic innervation of the frontal cortex, enhanced communication between fronto-parietal and fronto-temporal networks, and pubertal hormone changes that can alter brain function or neurotransmitter activity (e.g., Giorgio et al., 2010; Stiles and Jernigan, 2010). These physiological processes can strongly impact on ERP measures and topographies (Travis, 1998; Segalowitz et al., 2010) and could result in the observed ERP maps. Moreover, immature frontal processing could preclude the use of automatic strategies to perform a detection task and make the task harder for the youngest.

Therefore, the present study provides evidence for immature preparatory and resource allocation mechanisms supporting sustained attention in adolescents associated with frontal cortical development.

### Maturation of Predictive Processing

The present findings of a similar benefit in RT with predictive context irrespective of age suggest mature prediction capacities at age 12. This is in agreement with a previous work showing comparable amounts of commission errors in children and teenagers than in adults, in a detection task requiring motor inhibition to predictable non-target stimuli (McAvinue et al., 2012).

Moreover, we found that, irrespective of age, P3 amplitude progressively increases throughout the predictive sequence, i.e., as a function of task relevance and confidence (Sawaki and Katayama, 2006). In agreement with a role of the P3 in context-updating (Donchin and Coles, 1988), the present results support the notion that adolescents are able to extract predictive information from the stimulus train and to update working memory accordingly (Sawaki and Katayama, 2006; Fogelson et al., 2009b; Bidet-Caulet et al., 2012). Moreover, target predictability similarly shortens P3 latency and enhances CNV amplitude to the predicted targets in both adolescents and adults. A shortened P3 latency is ascribed to the shortened duration of stimulus evaluation processing (Kutas et al., 1977; Duncan-Johnson and Kopell, 1981) and an increased CNV response reflects the enhanced recruitment of preparatory mechanisms (Brunia and van Boxtel, 2001), confirming that prediction has been implemented. These predictability effects on the P3 latency and the CNV amplitude to targets correlate with the predictability benefit in reaction time, further supporting the use of P3 and CNV responses as markers of prediction abilities.

The present behavioral and electrophysiological results indicate mature anticipatory mechanisms (such as motor preparation), predictive information encoding/memorization and prediction building/implementation in adolescents at 12 year-old.

### Distinct Maturation Trajectories for Sustained Attention and Prediction

Attention and prediction are tightly entangled processes involved in processing optimization, which both strongly depend on the frontal activity (Corbetta et al., 2008; Bar, 2009; Summerfield and Egner, 2009). However, the present results show distinct brain maturation trajectories for attentive and predictive processing.

On one hand, goal-directed attention has been found to be mediated by a network principally including the lateral frontal cortex and posterior parietal regions (e.g., Corbetta and Shulman, 2002; Kastner and Pinsk, 2004; for reviews). Importantly, sustained attention requires the activation of this network to be maintained during the entire task duration (Sakai and Passingham, 2006). On the other hand, context-based associative predictions would be enabled by a cortical network which includes the medial temporal lobe, the medial parietal cortex and the medial frontal cortex (Bar, 2007, 2009), and also the lateral prefrontal cortex (Barcelo and Knight, 2007; Fogelson et al., 2009a). According to Friston’s predictive coding model (Friston, 2005), predictive processing is inherent to all processing levels of the hierarchically organized neural system, where top-down expectations and bottom-up stimulus information are integrated (Rao and Ballard, 1999; Friston, 2005). In the present study, target prediction could result from comparison between the visual input and a memory trace of the predictive sequence embodied in top-down predictions. If the generation of this memory trace most likely requires the activation of the frontal cortex, its maintenance could be achieved at another level of the hierarchy, (e.g., in visual areas).

Therefore, the differences in developmental trajectories for sustained attention and prediction during adolescence, characterized by protracted maturation of the frontal lobes, could be related to a larger and longer recruitment of the frontal cortex when attention is sustained than during simple predictive processing.

A limitation of this study is that the gender distribution is biased towards males in the three subsamples. This study would have also been stronger with larger samples, yet the present results seem quite clear given the rather strong power of the effects and the large effect sizes obtained (as defined by Cohen, 1992 and Richardson, 2011).

We demonstrated that predictive and attentive processing have different brain developmental trajectories after age 12. The present findings provide evidence for major developmental changes, from late adolescence to early adulthood, in preparatory and resource allocation mechanisms necessary to sustained attention, associated with frontal cortical development. Conversely, young adolescents seem to extract predictive information and use them to generate and implement simple prediction, as well as adults, suggesting mature predictive processing at the age of 12.

These findings raise important issues in the diagnosis and treatment of impairments in adolescents who suffer from neurodevelopmental disorders. The protracted maturation of sustained attention processes during adolescence stresses the importance of continued monitoring of teenagers with Attention Deficit Hyperactivity Disorder (Klein et al., 2012). The finding of mature prediction abilities in 12 year-olds highlights the importance of early diagnosis and intervention in Autism Spectrum Disorder, associated with difficulties in predictive processing (Gomot and Wicker, 2012).

## Funding

This work was supported by the Fondation Orange (AT), and NINDS grant R37NS21135 (RTK) and the Nielson Corporation. This work was performed within the framework of the LABEX CORTEX (ANR-11-LABX-0042) of Université de Lyon, within the program “Investissements d’Avenir” (ANR-11-IDEX-0007) operated by the French National Research Agency (ANR).

## Conflict of Interest Statement

The authors declare that the research was conducted in the absence of any commercial or financial relationships that could be construed as a potential conflict of interest.

## References

[B1] Aguera P. E. Jerbi K. Caclin A. Bertrand O. (2011). ELAN: a software package for analysis and visualization of MEG, EEG and LFP signals. Comput. Intell. Neurosci. 2011:158970. 10.1155/2011/158970 21687568 PMC3113286

[B2] Anderson V. A. Anderson P. Northam E. Jacobs R. Catroppa C. (2001). Development of executive functions through late childhood and adolescence in an australian sample. Dev. Neuropsychol. 20, 385–406. 10.1207/s15326942dn2001_5 11827095

[B3] Andrews-Hanna J. R. Mackiewicz Seghete K. L. Claus E. D. Burgess G. C. Ruzic L. Banich M. T. (2011). Cognitive control in adolescence: neural underpinnings and relation to self-report behaviors. PLoS One 6:e21598. 10.1371/journal.pone.0021598 21738725 PMC3125248

[B4] Bar M. (2007). The proactive brain: using analogies and associations to generate predictions. Trends Cogn. Sci. 11, 280–289. 10.1016/j.tics.2007.05.005 17548232

[B5] Bar M. (2009). The proactive brain: memory for predictions. Philos. Trans. R. Soc. Lond. B Biol. Sci. 364, 1235–1243. 10.1098/rstb.2008.0310 19528004 PMC2666710

[B6] Barcelo F. Knight R. T. (2007). An information-theoretical approach to contextual processing in the human brain: evidence from prefrontal lesions. Cereb. Cortex 17, i51–i60. 10.1093/cercor/bhm111 17726004

[B7] Becker D. E. Shapiro D. (1980). Directing attention toward stimuli affects the P300 but not the orienting response. Psychophysiology 17, 385–389. 10.1111/j.1469-8986.1980.tb00168.x 7394134

[B8] Bender S. Weisbrod M. Bornfleth H. Resch F. Oelkers-Ax R. (2005). How do children prepare to react? Imaging maturation of motor preparation and stimulus anticipation by late contingent negative variation. NeuroImage 27, 737–752. 10.1016/j.neuroimage.2005.05.020 16027009

[B9] Bidet-Caulet A. Barbe P. G. Roux S. Viswanath H. Barthélémy C. Bruneau N.. (2012). Dynamics of anticipatory mechanisms during predictive context processing. Eur. J. Neurosci. 36, 2996–3004. 10.1111/j.1460-9568.2012.08223.x 22780698 PMC3463677

[B10] Boelema S. R. Harakeh Z. Ormel J. Hartman C. A. Vollebergh W. A. M. van Zandvoort M. J. E. (2014). Executive functioning shows differential maturation from early to late adolescence: longitudinal findings from a TRAILS study. Neuropsychology 28, 177–187. 10.1037/neu0000049 24364395

[B11] Braver T. S. (2012). The variable nature of cognitive control: a dual mechanisms framework. Trends Cogn. Sci. 16, 106–113. 10.1016/j.tics.2011.12.010 22245618 PMC3289517

[B12] Brunia C. H. van Boxtel G. J. (2001). Wait and see. Int. J. Psychophysiol. 43, 59–75. 10.1016/S0167-8760(01)00179-9 11742685

[B13] Bubic A. von Cramon D. Y. Schubotz R. I. (2010). Prediction, cognition and the brain. Front. Hum. Neurosci. 4:25. 10.3389/fnhum.2010.00025 20631856 PMC2904053

[B14] Chatham C. H. Frank M. J. Munakata Y. (2009). Pupillometric and behavioral markers of a developmental shift in the temporal dynamics of cognitive control. Proc. Natl. Acad. Sci. U S A 106, 5529–5533. 10.1073/pnas.0810002106 19321427 PMC2666994

[B15] Chevalier N. James T. D. Wiebe S. A. Nelson J. M. Espy K. A. (2014). Contribution of reactive and proactive control to children’s working memory performance: insight from item recall durations in response sequence planning. Dev. Psychol. 50, 1999–2008. 10.1037/a0036644 24773104 PMC4222582

[B16] Cohen J. (1992). Statistical power analysis. Curr. Dir. Psychol. Sci. 1, 98–101.

[B17] Conners C. K. Epstein J. N. Angold A. Klaric J. (2003). Continuous performance test performance in a normative epidemiological sample. J. Abnorm. Child Psychol. 31, 555–562. 10.1023/A:1025457300409 14561062

[B18] Corbetta M. Shulman G. L. (2002). Control of goal-directed and stimulus-driven attention in the brain. Nat. Rev. Neurosci. 3, 201–215. 10.1038/nrn755 11994752

[B19] Corbetta M. Patel G. Shulman G. L. (2008). The reorienting system of the human brain: from environment to theory of mind. Neuron 58, 306–324. 10.1016/j.neuron.2008.04.017 18466742 PMC2441869

[B20] Courchesne E. Courchesne R. Y. Hillyard S. A. (1978). The effect of stimulus deviation on P3 waves to easily recognized stimuli. Neuropsychologia 16, 189–199. 10.1016/0028-3932(78)90106-9 692843

[B21] Donchin E. Coles M. G. H. (1988). Is the P300 component a manifestation of context updating? Behav. Brain Sci. 11, 357–374. 10.1017/s0140525x00058027

[B22] Duncan-Johnson C. C. Kopell B. S. (1981). The stroop effect: brain potentials localize the source of interference. Science 214, 938–940. 10.1126/science.7302571 7302571

[B23] Edgington E. S. (1995). Randomization Tests. New York: Marcel Dekker.

[B24] Fogelson N. Shah M. Bonnet-Brilhault F. Knight R. T. (2010). Electrophysiological evidence for aging effects on local contextual processing. Cortex 46, 498–506. 10.1016/j.cortex.2009.05.007 19559410 PMC2826523

[B25] Fogelson N. Shah M. Scabini D. Knight R. T. (2009a). Prefrontal cortex is critical for contextual processing: evidence from brain lesions. Brain 132, 3002–3010. 10.1093/brain/awp230 19713281 PMC2768662

[B26] Fogelson N. Wang X. Lewis J. Kishiyama M. Ding M. Knight R. (2009b). Multi-modal effects of local context on target detection: evidence from P3b. J. Cogn. Neurosci. 21, 1680–1692. 10.1162/jocn.2009.21071 18702574 PMC2823841

[B27] Friston K. (2005). A theory of cortical responses. Philos. Trans. R. Soc. Lond. B Biol. Sci. 360, 815–836. 10.1098/rstb.2005.1622 15937014 PMC1569488

[B28] Giorgio A. Watkins K. E. Chadwick M. James S. Winmill L. Douaud G.. (2010). Longitudinal changes in grey and white matter during adolescence. NeuroImage 49, 94–103. 10.1016/j.neuroimage.2009.08.003 19679191

[B29] Gomot M. Wicker B. (2012). A challenging, unpredictable world for people with autism spectrum disorder. Int. J. Psychophysiol. 83, 240–247. 10.1016/j.ijpsycho.2011.09.017 21968196

[B30] Greenberg L. M. Waldman I. D. (1993). Developmental normative data on the test of variables of attention (T.O.V.A. TM). J. Child Psychol. Psychiatry 34, 1019–1030. 10.1111/j.1469-7610.1993.tb01105.x 8408366

[B31] Hämmerer D. Li S.-C. Müller V. Lindenberger U. (2010). An electrophysiological study of response conflict processing across the lifespan: assessing the roles of conflict monitoring, cue utilization, response anticipation and response suppression. Neuropsychologia 48, 3305–3316. 10.1016/j.neuropsychologia.2010.07.014 20638396

[B32] Huttenlocher P. R. Dabholkar A. S. (1997). Regional differences in synaptogenesis in human cerebral cortex. J. Comp. Neurol. 387, 167–178. 10.1002/(sici)1096-9861(19971020)387:2<167::aid-cne1>3.0.co;2-z 9336221

[B33] Johnson R. Jr. (1986). A triarchic model of P300 amplitude. Psychophysiology 23, 367–384. 10.1111/j.1469-8986.1986.tb00649.x 3774922

[B34] Jonkman L. M. (2006). The development of preparation, conflict monitoring and inhibition from early childhood to young adulthood; a Go/Nogo ERP study. Brain Res. 1097, 181–193. 10.1016/j.brainres.2006.04.064 16729977

[B35] Kanaka N. Matsuda T. Tomimoto Y. Noda Y. Matsushima E. Matsuura M.. (2008). Measurement of development of cognitive and attention functions in children using continuous performance test. Psychiatry Clin. Neurosci. 62, 135–141. 10.1111/j.1440-1819.2008.01746.x 18412834

[B36] Kastner S. Pinsk M. A. (2004). Visual attention as a multilevel selection process. Cogn. Affect. Behav. Neurosci. 4, 483–500. 10.3758/cabn.4.4.483 15849892

[B37] Klein R. G. Mannuzza S. Olazagasti M. A. R. Roizen E. Hutchison J. A. Lashua E. C.. (2012). Clinical and functional outcome of childhood attention-deficit/hyperactivity disorder 33 years later. Arch. Gen. Psychiatry 69, 1295–1303. 10.1001/archgenpsychiatry.2012.271 23070149 PMC3597443

[B38] Knight R. T. Grabowecky M. F. Scabini D. (1995). “Role of human prefrontal cortex in attention control,” in Epilepsy and the Functional Anatomy of the Frontal Lobe Advances in Neurology (Vol. 66), eds. Riggio H. H. Jasper S. Goldman-Rakic P. S. (New York, NY, US: Raven Press), 21–36. 7771302

[B39] Kropp P. Linstedt U. Niederberger U. Gerber W. (2001). Contingent negative variation and attentional performance in humans. Neurol. Res. 23, 647–650. 10.1179/016164101101198956 11547936

[B40] Kutas M. McCarthy G. Donchin E. (1977). Augmenting mental chronometry: the P300 as a measure of stimulus evaluation time. Science 197, 792–795. 10.1126/science.887923 887923

[B41] Li S.-C. Hämmerer D. Müller V. Hommel B. Lindenberger U. (2009). Lifespan development of stimulus-response conflict cost: similarities and differences between maturation and senescence. Psychol. Res. 73, 777–785. 10.1007/s00426-008-0190-2 19023594 PMC2847161

[B42] Lin C. C. H. Hsiao C. K. Chen W. J. (1999). Development of sustained attention assessed using the continuous performance test among children 6–15 years of age. J. Abnorm. Child Psychol. 27, 403–412. 10.1023/A:1021932119311 10582841

[B43] Lucenet J. Blaye A. (2014). Age-related changes in the temporal dynamics of executive control: a study in 5- and 6-year-old children. Front. Psychol. 5:831. 10.3389/fpsyg.2014.00831 25120523 PMC4114259

[B44] Luna B. Garver K. E. Urban T. A. Lazar N. A. Sweeney J. A. (2004). Maturation of cognitive processes from late childhood to adulthood. Child Dev. 75, 1357–1372. 10.1111/j.1467-8624.2004.00745.x 15369519

[B45] McAvinue L. P. Habekost T. Johnson K. A. Kyllingsbæk S. Vangkilde S. Bundesen C.. (2012). Sustained attention, attentional selectivity and attentional capacity across the lifespan. Atten. Percept. Psychophys. 74, 1570–1582. 10.3758/s13414-012-0352-6 22825931

[B46] McCarthy G. Wood C. C. (1985). Scalp distributions of event-related potentials: an ambiguity associated with analysis of variance models. Electroencephalogr. Clin. Neurophysiol. 62, 203–208. 10.1016/0168-5597(85)90015-2 2581760

[B47] Mueller V. Brehmer Y. von Oertzen T. Li S.-C. Lindenberger U. (2008). Electrophysiological correlates of selective attention: a lifespan comparison. BMC Neurosci. 9:18. 10.1186/1471-2202-9-18 18237433 PMC2270855

[B48] Munakata Y. Snyder H. R. Chatham C. H. (2012). Developing cognitive control: three key transitions. Curr. Dir. Psychol. Sci. 21, 71–77. 10.1177/0963721412436807 22711982 PMC3375849

[B49] Padilla M. L. Pfefferbaum A. Sullivan E. V. Baker F. C. Colrain I. M. (2013). Dissociation of preparatory attention and response monitoring maturation during adolescence. Clin. Neurophysiol. 125, 962–970. 10.1016/j.clinph.2013.10.012 24211003 PMC3981931

[B50] Peltz C. B. Gratton G. Fabiani M. (2011). Age-related changes in electrophysiological and neuropsychological indices of working memory, attention control and cognitive flexibility. Front. Psychol. 2:190. 10.3389/fpsyg.2011.00190 21887150 PMC3157891

[B51] Perrin F. Pernier J. Bertrand O. Echallier J. F. (1989). Spherical splines for scalp potential and current density mapping. Electroencephalogr. Clin. Neurophysiol. 72, 184–187. 10.1016/0013-4694(89)90180-6 2464490

[B52] Polich J. (2007). Updating P300: an integrative theory of P3a and P3b. Clin. Neurophysiol. 118, 2128–2148. 10.1016/j.clinph.2007.04.019 17573239 PMC2715154

[B53] Rao R. P. N. Ballard D. H. (1999). Predictive coding in the visual cortex: a functional interpretation of some extra-classical receptive-field effects. Nat. Neurosci. 2, 79–87. 10.1038/4580 10195184

[B54] Richardson J. T. E. (2011). Eta squared and partial eta squared as measures of effect size in educational research. Educ. Res. Rev. 6, 135–147. 10.1016/j.edurev.2010.12.001

[B55] Rubia K. Hyde Z. Halari R. Giampietro V. Smith A. (2010). Effects of age and sex on developmental neural networks of visual-spatial attention allocation. NeuroImage 51, 817–827. 10.1016/j.neuroimage.2010.02.058 20188841

[B56] Rueda M. R. Posner M. I. (2013). “Development of attention networks,” in The Oxford Handbook of Developmental Psychology (Vol. 1), ed. Zelazo Philip David (New York: Oxford University Press), 683–705.

[B57] Sakai K. Passingham R. E. (2006). Prefrontal set activity predicts rule-specific neural processing during subsequent cognitive performance. J. Neurosci. 26, 1211–1218. 10.1523/jneurosci.3887-05.2006 16436608 PMC6674561

[B58] Sangal R. B. Sangal J. M. Belisle C. (1998). P300 latency and age: a quadratic regression explains their relationship from age 5 to 85. Clin. Electroencephalogr. 29, 1–6. 10.1177/155005949802900105 9472418

[B59] Sarter M. Givens B. Bruno J. P. (2001). The cognitive neuroscience of sustained attention: where top-down meets bottom-up. Brain Res. Brain Res. Rev. 35, 146–160. 10.1016/s0165-0173(01)00044-3 11336780

[B60] Sawaki R. Katayama J. (2006). Stimulus context determines whether non-target stimuli are processed as task-relevant or distractor information. Clin. Neurophysiol. 117, 2532–2539. 10.1016/j.clinph.2006.06.755 17005448

[B61] Segalowitz S. J. Davies P. L. (2004). Charting the maturation of the frontal lobe: an electrophysiological strategy. Brain Cogn. 55, 116–133. 10.1016/s0278-2626(03)00283-5 15134847

[B62] Segalowitz S. J. Dywan J. Unsal A. (1997). Attentional factors in response time variability after traumatic brain injury: an ERP study. J. Int. Neuropsychol. Soc. 3, 95–107. 9126851

[B63] Segalowitz S. J. Santesso D. L. Jetha M. K. (2010). Electrophysiological changes during adolescence: a review. Brain Cogn. 72, 86–100. 10.1016/j.bandc.2009.10.003 19914761

[B64] Smith A. B. Halari R. Giampetro V. Brammer M. Rubia K. (2011). Developmental effects of reward on sustained attention networks. NeuroImage 56, 1693–1704. 10.1016/j.neuroimage.2011.01.072 21300162

[B65] Stige S. Fjell A. M. Smith L. Lindgren M. Walhovd K. B. (2007). The development of visual P3a and P3b. Dev. Neuropsychol. 32, 563–584. 10.1080/87565640701361096 17650994

[B66] Stiles J. Jernigan T. L. (2010). The basics of brain development. Neuropsychol. Rev. 20, 327–348. 10.1007/s11065-010-9148-4 21042938 PMC2989000

[B67] Summerfield C. Egner T. (2009). Expectation (and attention) in visual cognition. Trends Cogn. Sci. 13, 403–409. 10.1016/j.tics.2009.06.003 19716752

[B68] Tamnes C. K. Fjell A. M. Westlye L. T. Østby Y. Walhovd K. B. (2012). Becoming consistent: developmental reductions in intraindividual variability in reaction time are related to white matter integrity. J. Neurosci. 32, 972–982. 10.1523/JNEUROSCI.4779-11.2012 22262895 PMC6621149

[B69] Tau G. Z. Peterson B. S. (2009). Normal development of brain circuits. Neuropsychopharmacology 35, 147–168. 10.1038/npp.2009.115 19794405 PMC3055433

[B70] Tecce J. J. (1971). Contingent negative variation and individual differences. A new approach in brain research. Arch. Gen. Psychiatry 24, 1–16. 10.1001/archpsyc.1971.01750070003001 4923214

[B71] Timsit-Berthier M. Hausmann J. (1972). [Study of the CNV and of the phenomenon of motor preparation in 5–15-year-old children]. Rev. Electroencephalogr. Neurophysiol. Clin. 2, 124–136. 4681588

[B72] Travis F. (1998). Cortical and cognitive development in 4th, 8th and 12th grade students: the contribution of speed of processing and executive functioning to cognitive development. Biol. Psychol. 48, 37–56. 10.1016/s0301-0511(98)00005-2 9676358

[B73] Yakovlev P. A. Lecours I. R. (1967). “The myelogenetic cycles of regional maturation of the brain,” in Regional Development of the Brain in Early Life, ed. Minkowski A. (Oxford: Blackwell), 3–70.

